# Comparing the impact on COVID‐19 mortality of self‐imposed behavior change and of government regulations across 13 countries

**DOI:** 10.1111/1475-6773.13688

**Published:** 2021-06-28

**Authors:** Julian C. Jamison, Donald Bundy, Dean T. Jamison, Jacob Spitz, Stéphane Verguet

**Affiliations:** ^1^ Economics Department University of Exeter Exeter UK; ^2^ Department of Disease Control London School of Hygiene & Tropical Medicine London UK; ^3^ Institute for Global Health Sciences University of California at San Francisco San Francisco California USA; ^4^ The World Bank Washington District of Columbia USA; ^5^ Department of Global Health and Population Harvard T.H. Chan School of Public Health Boston Massachusetts USA

**Keywords:** lockdown, nonpharmaceutical interventions, salience, SARS‐CoV‐2, voluntary behavior change, Western Europe

## Abstract

**Objective:**

Countries have adopted different approaches, at different times, to reduce the transmission of coronavirus disease 2019 (COVID‐19). Cross‐country comparison could indicate the relative efficacy of these approaches. We assess various nonpharmaceutical interventions (NPIs), comparing the effects of voluntary behavior change and of changes enforced via official regulations, by examining their impacts on subsequent death rates.

**Data Sources:**

Secondary data on COVID‐19 deaths from 13 European countries, over March–May 2020.

**Study Design:**

We examine two types of NPI: the introduction of government‐enforced closure policies and self‐imposed alteration of individual behaviors in the period prior to regulations. Our proxy for the latter is Google mobility data, which captures voluntary behavior change when disease salience is sufficiently high. The primary outcome variable is the rate of change in COVID‐19 fatalities per day, 16–20 days after interventions take place. Linear multivariate regression analysis is used to evaluate impacts.

Data collection/extraction methods: publicly available.

**Principal Findings:**

Voluntarily reduced mobility, occurring prior to government policies, decreases the percent change in deaths per day by 9.2 percentage points (pp) (95% confidence interval [CI] 4.5–14.0 pp). Government closure policies decrease the percent change in deaths per day by 14.0 pp (95% CI 10.8–17.2 pp). Disaggregating government policies, the most beneficial for reducing fatality, are intercity travel restrictions, canceling public events, requiring face masks in some situations, and closing nonessential workplaces. Other sub‐components, such as closing schools and imposing stay‐at‐home rules, show smaller and statistically insignificant impacts.

**Conclusions:**

NPIs have substantially reduced fatalities arising from COVID‐19. Importantly, the effect of voluntary behavior change is of the same order of magnitude as government‐mandated regulations. These findings, including the substantial variation across dimensions of closure, have implications for the optimal targeted mix of government policies as the pandemic waxes and wanes, especially given the economic and human welfare consequences of strict regulations.

**What is known on this topic?**

- Along with epidemiological data, analysts have tracked and published accounts of the nature, timing, and magnitude of government‐mandated nonpharmaceutical interventions (NPIs) for many countries.
- A substantial literature provides initial evidence on which NPIs do and which do not constructively affect the course of the pandemic, for example, typically international travel restrictions appear to do so but stay‐at‐home orders do not as much.
- Much less analysis has addressed the extent to which voluntary behavior change also has an important role to play in the response to the pandemic.

**What this study adds?**

- The pandemic in Europe led people to substantially reduce their own risky behavior, resulting in reduction of COVID‐19 mortality by an amount close to that of mandated NPIs.
- This suggests the value of government policies that enable or encourage voluntary NPIs (e.g., provision of free masks), as opposed to mandated NPIs (e.g., strict stay‐at‐home orders) which have a smaller benefit–cost ratio.
- Attributing the large adverse indirect economic consequences of the pandemic primarily to the government response would overstate the negative impact of government policy, given that an important component of diminished activity results from voluntary NPI.

## 1 INTRODUCTION

Over the course of 1 year, the transmission of the coronavirus disease 2019 (COVID‐19) has spread to essentially every country on the planet: as of June 2021, COVID‐19 has infected hundreds of millions of individuals and killed more than 3.5 million.^1^ During the first months of the pandemic, in the absence of available effective biomedical interventions like vaccines and treatments and in anticipation of an unprecedented surge of patients in need of intensive care in hospitals, a large number of national responses focused on the implementation of drastic nonpharmaceutical interventions (NPIs), including the closing of schools and universities, the prohibition of most commercial business, and the legal enforcement of local lockdowns and “shelter‐in‐place” orders. As a result, in May/June 2020, an estimated 1.2 billion children who should have been attending schools were not doing so,^2^ with long‐term consequences for learning potential and the creation of national capital, and hundreds of millions of adults have had to cease their economic activities, with profound and immediate consequences for national economies and personal livelihoods and well‐being. This is much more than a global health crisis.^3^

After the “first wave” of the epidemic receded in Western Europe, countries began to retrospectively examine their NPI policies, partly to assess when and how to reverse the school closure and movement restriction policies that have such substantial developmental and economic consequences, and partly to plan for subsequent epidemic waves. The challenge, however, is that the method used to originally select the NPIs may be less helpful for actual evaluation. In the absence of real data or prior experience, the evidence base supporting the rollout of such unprecedented NPIs relied on mathematical forecasting models^4^, ^5^, ^6^, ^7^, ^8^, ^9^ drawing on input parameters for epidemiologic quantities like severity and attack rate, risk factors, and timing of transmission, for which empirical validation remains nascent.^10^ These assumptions may have been inadvertently misleading, hence needing careful reassessment before being used as the basis for future decisions. For instance, with respect to school closures, a review of evidence from before COVID‐19^11^ as well as preliminary findings from Australia,^12^ France,^13^, ^14^ and Ireland^15^ suggest that school children—especially at primary level—may not be important drivers of coronavirus epidemics, in contrast to influenza, and school closure might play a substantially smaller role than the models had projected.

The need now is to retrospectively assess the true impact of NPIs on COVID‐related morbidity and mortality, in order to optimize their implementation (or lack thereof) going forward, using empirical evidence. In this respect, a number of studies have conducted retrospective analyses of the possible mitigating effects of NPIs on the COVID death toll at the country level or comparatively across countries.^8^, ^9^, ^16^, ^17^, ^18^, ^19^, ^20^, ^21^, ^22^, ^23^, ^24^, ^25^, ^26^ In particular, using a combination of modeling approaches, Haug and colleagues^21^ estimated the effectiveness of NPIs on the effective reproduction number across 56 countries and 79 territories and pointed out that less disruptive NPIs might be as effective as more drastic NPIs like national lockdowns. Likewise, Brauner et al.^20^ examined 34 European and seven non‐European countries and inferred that closing all educational institutions (in particular, including secondary and higher education), limiting gatherings to 10 people or less, and closing face‐to‐face businesses, each reduced transmission considerably.

In this paper, we use a time series of COVID‐related mortality data, over March–May 2020 during the first epidemic wave, from 13 comparable Western European countries to undertake a statistical examination of the timing of introduction of NPIs and their impact on daily COVID deaths. Crucially, we include not only the full spectrum of government‐mandated regulations but also proxy measures of voluntary behavior change before the introduction of the government policies. Here, “voluntary” simply means in the absence of government regulations or enforcement; the impetus may still arise from government or other institutional sources, in addition to peer effects (including social media) and purely self‐motivated change. This allows us to directly compare the potential effects of naturally salient social distancing and enhanced hygiene practices versus externally imposed and enforced regulations, with a view to contributing to the ongoing debate regarding restrictions on gatherings and movement; school and workplace closures; and other dimensions of government intervention in Europe and beyond.

## 2 METHODS

We conduct a statistical analysis of the potential impact of NPIs, either government‐imposed policies or voluntary behavior changes (before introduction of government policies), on COVID‐19 deaths over March–May 2020 among 13 Western European countries.

### 2.1 COVID‐19 mortality data

Daily figures for new confirmed COVID‐19 deaths by country were accessed through the European Centre for Disease Prevention and Control.^27^ We used data for the 13 Western European countries with greater than 500 COVID deaths as of 16 May (Table 1), all of which had 7–11 weeks of data, starting with date t_0_ which is defined when the 5‐day moving average of daily deaths is first equal to at least five. This March–May time‐period captures all of the closure policies but none of the subsequent relaxation of guidelines—where government and voluntary impacts are more difficult to disentangle.

**TABLE 1:** Summary statistics on COVID‐related deaths and timing of measures of voluntary behavior and government‐mandated regulations, for 13 European countries, March–May 2020

Country	Total number of deaths	Total number of deaths per million population	Date of *t*_0_	Date mobility falls	Date mobility fall impacts deaths	Date national stay‐at‐home restrictions announced	Date national stay‐at‐home restrictions impact deaths	Number of days of data
Austria	628	71	24 March	11 March	NA	6 March	22–26 March	56
Belgium	8959	784	19 March	12 March	28 March–1 April	18 March	3–7 April	61
Denmark	537	93	23 March	12 March	28 March–1 April	13 March	29 March–2 April	57
France	27 529	411	08 March	12 March	28 March–1 April	17 March	2–6 April	72
Germany	7881	95	18 March	9 March	NA	9 March	25–29 March	62
Ireland	1518	313	27 March	11 March	27–31 March	26 March	11–15 April	53
Italy	31 610	523	01 March	24 February	11–15 March	10 March	26–30 March	79
the Netherlands	5643	327	16 March	10 March	26–30 March	12 March	28 March–1 April	64
Portugal	1190	116	23 March	12 March	28 March–1 April	19 March	4–8 April	57
Spain	27 563	590	08 March	11 March	27–31 March	14 March	30 March–3 April	72
Sweden	3646	358	23 March	11 March	27–31 March	NA	NA	57
Switzerland	1594	187	19 March	10 March	26–30 March	17 March	2–6 April	61
United Kingdom	33 998	511	14 March	12 March	28 March–1 April	23 March	8–12 April	66

*Note*: Deaths data are cumulative up to and including 16 May. We describe policy changes as taking effect over a 5‐day date range because our model uses a 5‐day rolling average. Date *t*_0_ denotes the date at which the 5‐day rolling average reached five deaths, which we define as the start of the epidemic.

COVID mortality data were used because death constitutes a significant event; death certifications are less likely (than case notifications) to suffer from misclassifications; and the completeness of death data is far greater than that of case notification data due to varying testing capacity and accuracy across countries. However, (i) actual death tolls are still likely to differ from currently reported figures due to reporting issues, (ii) recording protocols can affect total numbers (e.g., whether deaths in nursing care homes are included), and (iii) reported date of death can be delayed from the actual date of death. Issues (i) and (ii) are mitigated here by focusing on relative changes in deaths, which also allow us to abstract away from total population size. Issue (iii) is mitigated in part by taking a 5‐day moving average of deaths. Hence, as our dependent variable, we study the evolution over time of the following percentage change in smoothed daily deaths:

(1) $${∆}_{i,t}=100*\frac{\sum_{k=1}^{5}d_{i,t+k-3}-\sum_{k=1}^{5}d_{i,t+k-4}}{\sum_{k=1}^{5}d_{i,t+k-4}},$$

where $d_{i,t}$ is the daily reported number of deaths in country *i* on day *t*.

To get a sense for the behavior of this variable ${∆}_{i,t}$, note that early in the pandemic, the number of deaths per day is typically rising, corresponding to the number of new infections having been growing a few weeks earlier, which implies that ${∆}_{i,t}>0$. Late in the pandemic, when the number of daily deaths is declining, this percent change will be negative. In between, each day will yield approximately the same number of deaths, and hence, our dependent variable will be around zero. Smaller values are always better, since they imply a slower rise in fatalities (if positive) or a more rapid decline (if negative). Table A1 in the Supplementary appendix shows the distribution of values of this variable in our data, week by week.

### 2.2 Nonpharmaceutical intervention data

For the interventions, we focus on two broad categories: government‐imposed policies and regulations vs self‐imposed and voluntary actions.

First, the Oxford COVID‐19 Government Response Tracker provides dates and intensities for multiple categories of government policies across the globe.^28^ Here, we focus on their “containment and closure” categories: school closing; workplace closing; canceling public events; restricting public events and gathering sizes; closing public transport; stay‐at‐home (or “shelter‐in‐place”) requirements; and restrictions on internal movement and international travel. Separately, we add information on facial coverings (including formal regulations) from their “health measures” category. We define two alternate independent variables of the government *closure* measure: (i) an easy‐to‐interpret *binary* closure measure (i.e., 0 or 1) that occurs whenever broad stay‐at‐home restrictions are first promulgated and (ii) a *continuous* closure measure which is the sum of scores across all included categories, normalized by dividing by the maximum such score in the database. That is, each country was given a score (0, 1, 2, 3, sometimes up to 4 or 5 depending on the category) at each point in time, reflecting the stringency of any regulations in effect. We add those scores across all of the categories listed above and then standardize so that the maximum possible value is 1, in order for interpretation to be comparable to the binary measure.

**FIGURE 1:**
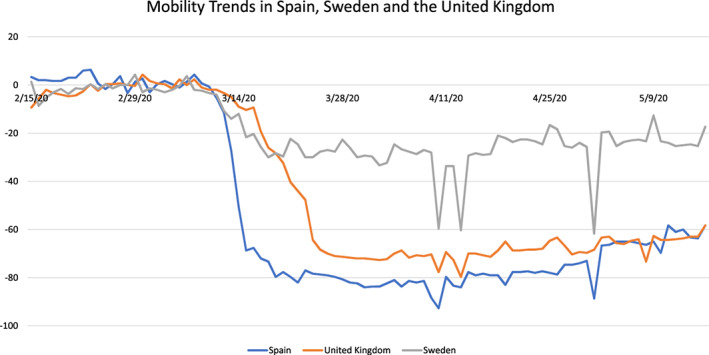
Change in mobility trends (February–May 2020) in Spain, Sweden, and the United Kingdom [Color figure can be viewed at wileyonlinelibrary.com]

Second, we also look at self‐imposed restrictions on behavior which arose prior to the introduction of governmental interventions. Our primary measure, *mobility decline*, is based on Google's Community Mobility Reports,^29^ which assess geographic mobility along different dimensions, as compared to a pre‐crisis baseline within each country. The aggregated anonymized data come from every mobile device for which a user has signed in to a Google account and turned on their “Location History” setting. We construct an independent variable (dummy indicator) that switches from 0 to 1 in a given country when the mobility index is negative (representing activity being below baseline levels) *and* remains so thereafter, for all of the following three mobility categories: workplaces, transit stations, and retail and recreation (see, for instance, Figure 1 which presents mobility data for three illustrative countries, aggregated across these three categories). We do not consider residential mobility (defined as time spent at one's primary location) nor grocery and pharmacy activity, since that involves essential activity. Similar changes were observed in China early in the pandemic, where regional air pollution, indicative of reduced traffic and production, decreased after cases were reported locally but before any government restrictions had been imposed.^30^

The mobility dummy indicator switches back from 1 to 0 when the government binary closure indicator turns on in that country because our goal is to evaluate the differential effect of unregulated behavior change. If binary closure takes place before self‐imposed mobility decline (as in Austria and Germany), then the mobility variable remains equal to 0 throughout the study period. As with closure, we also define a continuous version of this mobility‐independent variable equal to the normalized sum of mobility decline across the three relevant categories, on a given date. That is, we add up the percentage decreases across workplace, transit, and retail/recreation and then standardize so that the maximum value equals 1 and is comparable to the binary measure.

### 2.3 Statistical modeling approach

First of all, evidently, none of these interventions, either regulatory or voluntary, will have an immediate effect on fatalities due to COVID‐19. Rather, we hypothesize that they will change the rate of new infections, leading to a change in deaths some time later. In order to model that delay, we assume that it is the sum of the incubation period, estimated to be 5 days,^5^ and the period from symptom onset to death (for those who die), which has an observed median of 13 days.^31^ Note that the overall typical time to death will be different from 13 days because in a growing epidemic, proportionately, more observations are from recent infections (some of whom will die later). We are modeling the observed data in the mid of a growing epidemic; hence, it is precisely the raw data that we need to match. Thus, we assume a median lag of 18 days from time of intervention to time of death. There is naturally some distribution for this lag, thus we employ a 5‐day moving average of deaths, corresponding to lags from 16 to 20 days (Table 1).

**FIGURE 2:**
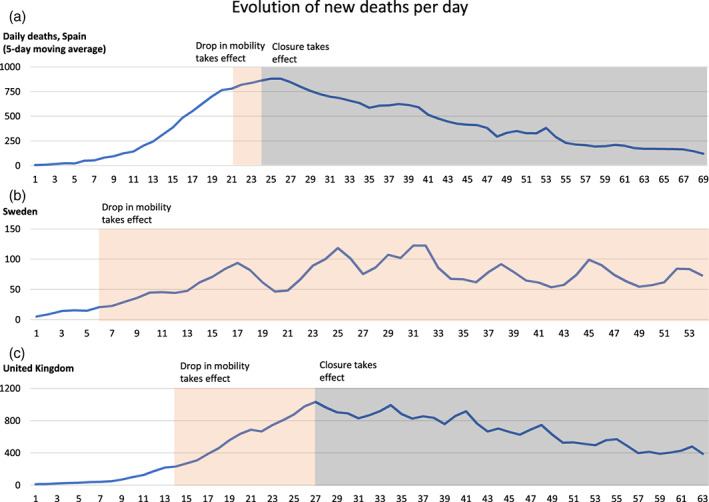
Evolution of the daily deaths since *t*_0_ (the date at which the 5‐day moving average reaches five deaths) in Spain, Sweden, and the United Kingdom [Color figure can be viewed at wileyonlinelibrary.com]

Figure 2 shows the 5‐day average deaths for three illustrative countries, along with the middle of the range of dates at which binary closure and (if relevant) binary mobility decline are assumed to have taken effect. The three countries include Spain and the United Kingdom, which both have relatively large populations, but where the local epidemic started relatively early and relatively late respectively, as well as Sweden, which is unique in our sample in that the government never imposed stay‐at‐home regulations.

Second, we evaluate the effect of NPIs (via our two independent variables that track government‐imposed policies [*Policy*] vs self‐imposed behavior changes [*Behavior*], as defined above) on the rate of change in COVID deaths via a linear regression model. The daily percentage change in deaths is our dependent variable (denoted ${∆}_{i,t}$), and we use a random effects specification to further net out a range of country‐specific factors that might affect both the total number of deaths and local behavioral and policy changes, such as the scale of the epidemic, political ideology, or infrastructure differences. We employ the following model specification for the daily percentage change in COVID deaths in country *i* at time *t*:

(2) $${∆}_{i,t}=\alpha +\beta_{1}{\text{Behavior}}_{i,\left(t-18\right)}+\beta_{2}{\text{Policy}}_{i,\left(t-18\right)}+\gamma X_{i}+\theta_{i}+\mu_{1}t+\mu_{2}t^{2}+e_{i,t}.\;$$

${\text{Behavior}}_{i,\left(t-18\right)}$ and ${\text{Policy}}_{i,\left(t-18\right)}$ are our indicators of behavioral and policy changes (as defined above), respectively. They are lagged by 18 days to reflect when policy changes materialize (i.e., delayed impact of intervention on COVID‐19 deaths), as detailed above. Meanwhile, *t* counts the number of days since the start of the epidemic in each country: this captures exogenous time trends, as well as technological innovation and any endogenous learning by both individuals and clinicians. Because this effect is expected to be nonlinear, we also include *t* ^2^ as an independent variable. As usual, $e_{i,t}$ is a mean zero exogenous error term.

$X_{i}$ is a vector of time‐invariant country‐specific controls: share of population older than 65,^32^ population density,^32^ the number of acute care beds per 100,000 population,^33^ as well as the starting date (*t*_0_) of the epidemic in each country (Table 1). These may each affect the severity of the outbreak independently of any interventions; however, the main goal here is to directly compare NPIs across as similar contexts as possible and hence to avoid any other observable confounds as much as possible. For robustness, we also included a specification adding relevant time‐varying country‐specific controls $X_{\mathrm{it}}$: the test positivity rate and either intensive‐care or total hospital patients^27^ (per million).

^a^Lastly, $\theta_{i}$ is a country‐specific variable; under the random effects model, we assume that conditional on country controls $X_{i}$; all other country‐specific variations are distributed randomly.

In addition, in order to help interpret the implications of the potential effect sizes of the interventions (i.e., $\beta_{1}$ and $\beta_{2}$ in Equation (2)), we estimated the number of days for deaths to double, as of 1 week into the epidemic, under the following three scenarios: (i) no intervention; (ii) closure only; and (iii) voluntary behavior change only. To do this, we fixed *t* = 7, predicted the expected country‐average growth rate ${\hat{\bar{∆}}}_{\left(t=7\right)}$ in each of the three scenarios respectively, and applied the following formula for doubling time: $\tau_{\text{double}}=\left(\ln\;\left(2\right)/\ln\left(1+{\hat{\bar{∆}}}_{\left(t=7\right)}\right)\right)$.

Finally, we estimated the number of COVID deaths that would have occurred in the first 7 weeks from the local starting date (*t*_0_), under analogous scenarios: (i) no intervention; (ii) closure only (starting at *t* = 7); and (iii) salience only (starting at *t* = 7). This was calculated by summing deaths from *t* = 0^b^ through *t* = 50, after iterating forward using the modeled growth rates (from Equation (2)) in each of the three hypothetical policy scenarios. Each country has slightly different values for the time‐invariant controls (such as age distribution and population density), which lead to different predicted growth rates above, but the primary cross‐country variation in our model comes from the local starting calendar date of the epidemic. Hence, we are not attempting to directly compare the efficacy of policy choices across countries; we are using the existing empirical variation to estimate what various possible responses would have looked like for a prototypical (European) country, conditional on when the pandemic initially hit.

All statistical analyses used STATA/SE version 13.0. All data come from publicly available aggregate sources, so no ethical approval was required. No external funding was utilized during the course of the study.

## 3 RESULTS

Table 2 presents the main results, with Model I being the preferred specification for ease of interpretation. The first row shows that as time passes, regardless of any external intervention, death rates go down: for each day that passes, the daily change in fatalities goes down by 0.88 (95% confidence interval: 0.67–1.10) percentage points (pp).

**TABLE 2:** Effect of observed mobility and government closure on daily change in deaths, for 13 European countries, March–May 2020

	Model I (18‐day lag)	Model II (17‐day lag)	Model III (19‐day lag)	Model IV (continuous)
Days from *t*_0_	−0.88***	−0.86***	−0.90***	−0.83***
	[−1.10, −0.67]	[−1.06, −0.67]	[−1.10, −0.70]	[−1.05, −0.61]
Days from *t*_0_‐squared	0.009***	0.008***	0.009***	0.008***
	[0.006, 0.011]	[0.006, 0.010]	[0.006, 0.11]	[0.006, 0.107]
Binary mobility	−9.2***	−10.2***	−9.0***	
	[−14.0, −4.5]	[−14.7, −5.7]	[−12.7, −5.4]	
Continuous mobility				−8.5*
				[−16.0, −1.1]
Binary closure	−14.0***	−15.2***	−13.1***	
	[−17.2, −10.8]	[−18.7, −11.7]	[−15.5, −10.7]	
Continuous closure				−22.1***
				[−27.4, −16.9]
Percent of population older than 65	−0.18	−0.17	−0.17	−0.13
	[−0.42, 0.06]	[−0.41, 0.06]	[−0.45, 0.10]	[−0.67, 0.41]
Population density	0.001	0.001	0.001	−0.003
	[−0.004, 0.007]	[−0.004, 0.007]	[−0.004, 0.006]	[−0.010, 0.004]
Number of acute care beds, per 100 000 people	0.002	0.002	0.002	0.004
	[−0.005, 0.009]	[−0.005, 0.009]	[−0.005, 0.008]	[−0.004, 0.012]
Date of *t*_0_	−0.21***	−0.20***	−0.21***	−0.34***
	[−0.28, −0.14]	[−0.27, −0.13]	[−0.29, −0.13]	[−0.45, −0.24]
Number of observations	778	778	778	776

*Note*: 95% Confidence intervals are presented in square brackets. Standard errors are clustered at the country level. The unit of observation is a country‐day: 1 day of data for a specific country. Observed mobility is the binary measure based off Google mobility data; continuous mobility is a measure calculated by summing the same three measures of the Google Mobility Index and normalizing across countries; binary closure is our binary variable based on the Oxford Policy Tracker index for stay‐at‐home restrictions; and continuous closure is a measure calculated by summing all eight “containment and closure” categories in the Oxford Policy Tracker and normalizing across countries. We make one small change to the Oxford data: defining the German lockdown as being nationwide instead of regional. N is lower in Model IV because the lagged mobility data are only available for Italy from the third day of the epidemic. Model IV assumes an average 18‐day lag for mortality, like Model I.

* Significant at 5% level.

** Significant at 1% level.

*** Significant at 0.1% level.

Both the voluntary measure (self‐imposed mobility) and the closure measure (government restrictions) have substantial impacts on death rates 18 days later. A binary reduction in self‐imposed mobility is associated with a 9.2 (4.5–14.0) pp reduction in the daily rate of change in fatalities, while binary government restrictions are associated with a 14.0 (10.8–17.2) pp reduction. This means that if deaths were initially *growing* by 5% per day, then voluntary behavior change would cause them to start *declining* by 4.2% per day instead, while government regulations would cause them to decline by 9% per day.

Models II and III estimate the same specification but with fatality lags of 17 and 19 days, respectively (instead of a lag of 18 days in Model I, which was our best estimate from the medical literature), confirming that the results are robust across modeling choices. Model IV shows that the continuous measures (in place of binary measures, both for voluntary mobility and for government restrictions) also yield similar results. The closure measure (government restrictions) in this case has a larger magnitude because taking on a value of 1 signifies (as defined) that all government policies are being enacted at the maximal observed level simultaneously. A typical policy change is less extreme and therefore corresponds to a proportionately reduced impact as in Models I–III. However, the main result—that is, both voluntary and regulatory NPIs significantly reduce mortality, with the latter having a somewhat larger impact—carries over in any case.

The Supplementary appendix reports further sensitivity analyses: (i) a specification of the main results with country fixed effects (Table A2), (ii) a specification of the main results controlling for real‐time health system capacity constraints such as intensive‐care patients per capita (Table A3), (iii) an alternate proxy for voluntary behavior change, occurring when the number of national deaths surpasses a salience threshold of 5 (Table A4 Model II), and (iv) forced equalization of the length of epidemic (so that each country has the same number of days of data in the model; Table A4 Model III). Alternative (i) produces very similar results, but a Hausman test selects the random effects model as the preferred primary specification. Alternative (ii) also leads to very similar results for the primary variables; the additional controls are not significant and reduce the sample size due to missing data for some countries. Alternative (iii) yields directionally similar results, with smaller magnitudes for both the voluntary (salience) and regulatory (closure) measures. This is unsurprising, since the simple salience indicator is necessarily coarser and more *ad hoc* than the mobility data. Alternative (iv) is again very similar to the primary specification, but by construction has fewer observations (so was not preferred).

Table 3 separately reports on the effect arising from different components when the continuous closure metric is used. Model I looks at all eight categories individually, finding that three of them are statistically significant. Closing nonessential workplaces is estimated to reduce the change in deaths by 4.0 (0.5–7.4) pp; restricting public events reduces it by 5.9 (2.0–9.8) pp; limiting international travel reduces it by 5.4 (1.1–9.6) pp; and requiring face masks in some public situations reduces it by 6.8 (2.2–11.3) pp. Subsequent columns combine qualitatively similar categories to estimate the effect of the corresponding policies, as well as to check for sensitivity to the particular definitions used in the country tracker data. Overall everything is robust, with the possible exception of limiting public events (which no longer appears effective when combined with restrictions on the size of gatherings).

**TABLE 3:** Disaggregated impact of the various nonpharmaceutical interventions on daily change in deaths, for 13 European countries, March–May 2020

	Model I	Model II	Model III	Model IV
School closure	−2.9	−3.3	−2.5	−22.1***
	[−6.4, 0.62]	[−7.0, 0.5]	[−6.1, 1.1]	
Workplace closure	−4.0*	−4.1*	−4.0	
	[−7.4, −0.5]	[−7.6, −0.5]	[−8.9, 0.9]	
Restricting events	−5.9**	−2.2	−13.0***	
	[−9.8, −2.0]			
Restricting gathering size	3.1**	[−7.6, 3.2]		
	[1.0, 5.2]			
Closure of public transport	2.5	−9.5*		
	[−1.7, 6.6]			[−27.4, −16.9]
Stay‐at‐home restrictions	−3.7		[−20.0, −6.1]	
	[−11.8, 4.4]			
Restrictions on internal travel	−2.5	[−16.9, −2.2]		
	[−7.3, 2.2]			
Restrictions on international travel	−5.4*			
	[−9.6, −1.1]			
Face mask requirements	−6.8**	−4.0*	−4.1*	
	[−11.3, −2.2]	[−7.8, −0.3]	[−7.6, −0.62]	
Continuous mobility	−7.5	−8.8*	−10.0*	−8.5*
	[−15.7, 0.62]	[−16.1, −1.4]	[−17.9, −2.1]	[−15.9, −1.1]
Number of observations	776	776	776	776

*Note*: 95% Confidence intervals are presented in square brackets. Specifications also included controls for *t*, *t*‐squared, the percentage of population older than 65, the population density, and number of acute care beds per 100,000 people, and the date when the 5‐day moving average of daily deaths is first equal to at least five. Standard errors are clustered at the country level. The unit of observation is a country‐day: 1 day of data for a specific country. All indicators of government restrictions are as defined in the Oxford tracker and are normalized across an interval [0,1] for the 13 countries. N lower than in Models I–III in Table 2 because the lagged mobility data are only available for Italy from the third day of the epidemic.

* Significant at 5% level.

** Significant at 1% level.

*** Significant at 0.1% level.

Furthermore, we use the coefficients in Model I (see Table 2) to estimate the number of days for deaths to double, as of *t* = 7, under three scenarios: (i) no intervention, (ii) closure only, and (iii) voluntary mobility reduction only. Doubling time is increased from 3.0 (2.7–3.4) to 4.5 (3.7–5.8) days with a voluntary reduction in mobility (salience scenario), or to 6.1 (5.0–8.1) days with government lockdown.

Finally, we compare our estimated projections of the number of deaths under scenarios (i), (ii), and (iii), compared with observations (since the epidemic start) from exemplar countries. For example, Spain would have had 174,935 deaths over the first 50 days of the epidemic if there had been no interventions at all, while in reality, 23,467 deaths were observed over that same period. Yet, if Spain had closed down 2 weeks earlier, deaths would have been only 3487. Furthermore, even with purely self‐imposed changes throughout, as long as those began equally early, the number of deaths would have been only 11,430. Thus, the timing of interventions is crucial: tens of thousands of lives could have been saved even without a full lockdown. Meanwhile, Sweden experienced a later epidemic start and, in the absence of any interventions, would have seen an estimated 53,528 deaths in its first 50 days. Rather, 3271 deaths were reported in Sweden over the same time period‐similar to our projection for scenario (iii) of voluntary changes only (since that was indeed what happened there, although the timing was slightly different)—while government closure could have reduced this further to 1494.

## 4 DISCUSSION

Using daily death count data from 13 European countries over March–May 2020, we find that NPIs could substantially reduce fatalities from COVID‐19. Both voluntary behavior change prior to official government guidelines, as well as strict government regulations themselves, had a significant effect on the evolution of the rate of change of deaths. The magnitudes of the two approaches were not markedly different from one another: government closures reduced the rate of change of deaths by around 14 pp, while behavior change (in the absence of government regulations) reduced it by around 9 pp. Either approach, if in place realistically early, would have saved thousands of lives in a typical country in our sample.

A number of previous observational analyses of NPIs, including primarily stay‐at‐home, social distancing directives, closing of educational institutions, closing of businesses, and limiting gatherings, have focused on estimating impact on cases and reproduction numbers, either in individual countries^24^, ^34^, ^35^, ^36^, ^37^, ^38^, ^39^ or across multiple countries.^20^, ^21^, ^22^, ^23^, ^26^, ^40^, ^41^ There is limited existing work regarding the effect specifically of stay‐at‐home policies and government interventions on deaths, for example, in Brazil,^42^ France,^16^ Sweden,^17^ the United States,^18^ and across countries.^19^ Similar to our paper, Flaxman et al.^8^ examined the impact of regulations on fatalities in 11 European countries. They assessed multiple government interventions (including explicit encouragement of social distancing) but did not consider voluntary behavior change as here. Their main result was that lockdowns had a strong impact, but surprisingly that no other policies (social distancing, limiting public events, closing schools, or self‐isolation) had a significant effect at all. Our conclusions are distinct, perhaps due to the fact that we take a naïve but direct statistical approach to the relationships rather than filtering them through a complex structural mathematical model.

Another publication^41^ studied the impact of multiple interventions on cases (not deaths) across six countries globally, using a similar reduced‐form approach to that taken here. However, like the other studies, it does not consider voluntary changes, so its counterfactual scenario (exponential growth of 38% per day in the absence of policy) becomes less and less realistic over time, exaggerating the role of explicit policies in flattening the curve. Lastly, Haug et al.^21^ modeled intervention impact on the effective reproduction number across 56 countries and showed that less disruptive interventions might be as effective compared to drastic ones (i.e., lockdowns). Brauner et al.,^20^ in analyzing 34 countries, concluded that closing all educational institutions, limiting gatherings, and closing businesses each substantially reduced COVID‐19 transmission.

To our knowledge, only a handful of previous studies, particularly in the context of the United States,^25^, ^39^, ^43^, ^44^, ^45^ have explored the remarkable impact of self‐imposed behavior change, also via mobility data, yet these empirical results suggest that enforced lockdown regulations offer only modestly stronger epidemiological outcomes than well‐timed voluntary behavior change. This is an important observation in the context of addressing future spikes or epidemics in other countries, especially given that self‐motivated behaviors (e.g., social distancing, improved personal hygiene, reducing unnecessary travel, and working remotely when possible) are intrinsically less disruptive and more individually malleable than regulatory options (e.g., shelter‐in‐place orders, closing schools, and banning public transit). Although we did not include the United States in our analysis, since our goal was to isolate the impacts of NPIs per se (rather than cross‐country comparisons), there is no reason to believe that the relative qualitative conclusions comparing interventions would not carry over, even if the magnitudes are not the same.

Our results also provide insights into which of the government regulations were more effective when we disaggregate government policies into various subcategories. Limiting travel, particularly international travel, seems to have a significant effect, as does closure of nonessential workplaces. However other categories, including closing schools and imposing stay‐at‐home rules, show smaller and statistically insignificant effects. These subcategory findings are to be interpreted with caution, in part because there is less variation than optimal across countries in our sample, but they do suggest that policy makers should think carefully about whether—and if so for how long—the various restrictions, some of which are known to inflict large costs on health, education, welfare, and the economy, are necessary.

As one of a few studies to explore these issues empirically using substantial data, our analysis inevitably involves a number of limitations. Without randomization or other exogenous variation in the treatments, evidently, we cannot fully ascertain a causal link between the NPIs and the resulting changes in death rates. We do not expect any direct reverse causality, since future deaths will not change current behaviors. Current case rates could impact both variables, although as long as that effect is similar across dimensions it will not change the relative performance between voluntary versus government NPIs, or within the latter, which is our main outcome of interest. It is conceivable that some third variable, for instance, heightened media attention and scrutiny, could directly influence both government policies and individual behaviors. Future studies, using data on this and similar potential confounders, may be able to fully disentangle the various mechanisms at play.

Beyond endogeneity concerns, the quality of the fatality data may be subject to variation in reporting standards across countries, although this will be mitigated for the most part by focusing on rates of change rather than levels. Similarly, the quality of the government closure data is, although compiled independently without any apparent bias, somewhat subjective in nature as to the precise degree of severity in each category at each point in time. Meanwhile the mobility data, while more objective, does not capture the full range of voluntary self‐protective behaviors (such as hand washing and maintaining personal distance). Our supposition is that these are all highly correlated with one another, but if this relationship differs substantially over time, then it could fail to be a good proxy for overall voluntary changes; it is not a priori clear in which direction this would affect the current results.

Our main messages are that NPIs can have significant impacts in reducing COVID‐19 mortality and that almost half of this effect arises from simpler and more flexible voluntary interventions such as micro‐level behavioral change, working remotely to the extent possible, and reducing discretionary travel, as opposed to stricter officially imposed regulations. Precisely, why that is we cannot say from our analysis—other research has examined, for example, sociodemographic differences^46^—but the distinction is clearly important to countries at any stage of responding to the pandemic. Indeed this was suggested in a paper as early as March 2020: “Personal, rather than government action, in western democracies might be the most important issue.”^47^ These lessons are relevant around the globe, although cost‐effective targeting and evidence‐based policy are likely even more important for resource‐constrained countries with a weaker health and financial safety net.

## Supporting information


**Appendix S1** Supplementary information

## Endnotes

^a^ We thank a reviewer for suggesting this.

b Recall *t*_0_ is defined when the 5‐day moving average of daily deaths is first equal to at least 5; for this counterfactual analysis, we normalized all countries to start at exactly 5 so that small changes in initial conditions (driven solely by the discrete nature of the threshold) would not arbitrarily affect the results.
