# Supplementary material for: Comparing the impact on COVID‐19 mortality of self‐imposed behavior change and of government regulations across 13 countries
Source: Health Serv Res. 2021 Jun 28;56(5):874–84. doi: 10.1111/1475-6773.13688 (PMC8441808; doi:10.1111/1475-6773.13688)
Supplement: Supplementary file 1 — Appendix S1 Supplementary information [file HESR-56-874-s001.docx]

**Supplementary Appendix**

| Table A1: Percentile distribution of daily change in smoothed deaths, by week | | | | | | | | |
| --- | --- | --- | --- | --- | --- | --- | --- | --- |
| **Percentile** | **Overall** | **Week 1** | **Week 2** | **Week 3** | **Week 4** | **Week 5** | **Week 6** | **Week 7** |
| **10th** | -15.9% | 0.0% | -1.8% | -7.6% | -9.6% | -16.5% | -17.8% | -22.7% |
| **25th** | -6.1% | 9.6% | 7.7% | -1.2% | -4.0% | -6.2% | -13.6% | -13.0% |
| **50th** | 1.5% | 21.6% | 15.4% | 8.1% | 0.4% | -2.0% | -4.6% | 5.2% |
| **75th** | 12.8% | 39.5% | 24.3% | 13.6% | 8.5% | 4.5% | 2.5% | 5.3% |
| **90th** | 25.4% | 57.1% | 32.3% | 18.6% | 16.3% | 10.8% | 9.3% | 16.7% |
| **Observations** | 778 | 91 | 91 | 91 | 91 | 91 | 91 | 91 |

The distribution of the 5-day average of deaths (Table A1) shows that the growth in deaths is monotonically decreasing over time, but less and less strongly as time passes, supporting the assumption of our linear plus quadratic specification. In week 1, mortality is increasing in all countries, after which it begins to decline for many but not all observations, and finally the downward trend seems to plateau by weeks 6-7.

| Table A2: Effect of Salience and Closure on Daily Change in Deaths using Country Fixed Effects | | | | |
| --- | --- | --- | --- | --- |
|  | **Model I**  **(18 day lag)** | **Model II**  **(17 day lag)** | **Model III**  **(19 day lag)** | **Model IV**  **(continuous)** |
| **Days from t_0_** | -0.96*** | -0.93*** | -0.99*** | -0.73*** |
|  | [-1.22, -0.71] | [-1.17, -0.70] | [-1.21, -0.77] | [-1.01, -0.45] |
| **Days from t_0_ squared** | 0.0094*** | 0.0091*** | 0.0097*** | 0.0070*** |
|  | [0.0067, 0.012] | [0.0067, 0.11] | [0.0074, 0.012] | [0.0042, 0.0098] |
| **Binary mobility** | -9.4** | -10.4*** | -9.2*** |  |
|  | [-15.0, -3.7] | [-15.9, -4.9] | [-13.7, -4.7] |  |
| **Continuous mobility** |  |  |  | -11.9* |
|  |  |  |  | [-21.5, -2.3] |
| **Binary closure** | -12.1*** | -13.6*** | -10.9*** |  |
|  | [-16.2, -8.1] | [-17.8, -9.4] | [-13.8, -8.0] |  |
| **Continuous closure** |  |  |  | -21.1*** |
|  |  |  |  | [-28.0, -14.3] |
| **Number of observations** | 778 | 778 | 778 | 776 |
| Notes: * = significant at 5% level, ** = significant at 1% level, *** = significant at 0.1% level, 95% confidence intervals are presented in square brackets. Specifications also included controls for *t, t* squared, the percentage of population aged over 65, the population density, and number of acute care beds per 100,000 people, and the date when the five-day moving average of daily deaths is first equal to at least five. Standard errors are clustered at the country level. N lower in Model IV because the lagged mobility data is only available for Italy from the third day of the epidemic. Model IV assumes an average 18-day lag for mortality, like Model I. | | | | |

Table A2 presents the results running Models I, II, III, and IV in Table 2 (in the main text) using a fixed effects specification:

$$\Delta_{i,t}=\alpha+\beta_{1}{Behavior}_{i,(t-18)}+\beta_{2}{Policy}_{i,(t-18)}+\theta_{i}+\mu_{1}t+\mu_{2}t^{2}+e_{i,t} . (A1)$$

The model includes a $\theta_{i}$term to capture aggregate country-specific variation, and excludes $X_{i}$ due to collinearity. However, while it does not require the random effects assumption, the fixed effects model is less efficient compared with the random effects model. To compare the two specifications, we run a Hausman test, without clustering standard errors, to compare the estimates in our fixed effects model to our random effects model with controls. The results confirm the random effects assumption, and so we use the random effects model as our primary specification.

| Table A3: Effect of controlling for severity of epidemic on model results | | | |
| --- | --- | --- | --- |
|  | **Model I** | **Model II** | **Model III** |
| **Binary mobility** | -11.7*** | -9.4*** | -8.7*** |
|  | [-17.4, -6.0] | [-14.6, -4.3] | [-14.1, -3.4] |
| **Binary closure** | -13.5*** | -14.0*** | -13.9*** |
|  | [-21.0, -6.0] | [-18.2, -9.9] | [-17.8, -10.0] |
| **Test positivity rate** | 14.4 |  |  |
|  | [-11.7, 40.4] |  |  |
| **ICU patients per million** |  | -0.02 |  |
|  |  | [-0.06, 0.02] |  |
| **Hospital patients per million** |  |  | -0.005 |
|  |  |  | [-0.015, 0.004] |
| **Number of observations** | 472 | 571 | 454 |
| Notes: * = significant at 5% level, ** = significant at 1% level, *** = significant at 0.1% level, 95% confidence intervals are presented in square brackets. Specifications also included controls for *t, t* squared, the percentage of population aged over 65, the population density, and number of acute care beds per 100,000 people, and the date when the five-day moving average of daily deaths is first equal to at least five. Standard errors are clustered at the country level. Observed mobility is the binary measure based off Google mobility data; continuous mobility is a measure calculated by summing the same three measures of the Google Mobility Index and normalizing across countries; binary closure is our binary variable based on the Oxford Policy Tracker index for stay-at-home restrictions. Model I drops France and Sweden due to missing data, Model II drops Spain and Switzerland, and Model III drops Germany, Netherlands, Spain and Switzerland. | | | |

Table A3 presents the results from adding additional controls for outbreak severity. If hospitals become overwhelmed, that can have an effect on the mortality rate that is separate to government policy and would need to be controlled for. We test three different controls – test positivity rate, ICU patients per million, and hospital patients per million – and find that none are significant in explaining the change in daily deaths, nor do they change the significance of binary mobility or binary closure. Including each control reduces the number of observations, due to missing data, and for this reason we do not include them in the main specification.

| Table A4: Substituting Inherent Salience for Observed Mobility and Equalizing Length of Data | | | |
| --- | --- | --- | --- |
|  | **Model I** | **Model II** | **Model III** |
| **Days from t_0_** | -0.88*** | -1.1*** | -1.06*** |
|  | [-1.1, -0.67] | [-1.3, -0.86] | [-1.6, -0.59] |
| **Days from t_0_ squared** | 0.0087*** | 0.011*** | 0.012** |
|  | [0.0062, 0.011] | [0.0085, 0.013] | [0.0035, 0.020] |
| **Binary mobility** | -9.2*** |  | -7.9*** |
|  | [-14.0, -4.5] |  | [-12.8, -3.1] |
| **Inherent salience** |  | -2.07 |  |
|  |  | [-7.0, 2.8] |  |
| **Binary closure** | -14.0*** | -8.3*** | -12.4*** |
|  | [-17.2, -10.8] | [-13.3, -3.3] | [-15.3, -9.4] |
| **Number of observations** | 778 | 778 | 650 |
| Notes: p* = significant at 5% level, ** = significant at 1% level, *** = significant at 0.1% level, 95% confidence intervals are presented in square brackets. Specifications also included controls for *t, t* squared, the percentage of population aged over 65, the population density, and number of acute care beds per 100,000 people, and the date when the five-day moving average of daily deaths is first equal to at least five. Standard errors are clustered at the country level. Model III limits the number of days of data in each regression to 50, which is the lowest total in the group of countries (Ireland). | | | |

Mobility decline has the advantage of relatively objectively measuring a subset of pertinent behavior, but it only captures a subset of the relevant aspects – in particular nothing to do with personal hygiene (e.g. handwashing) or with maintaining distance when interacting with others. Although we cannot directly measure these, a natural assumption is that they increase in prevalence when the pandemic becomes locally salient, approximated by the moment when the average daily number of deaths reaches 5 in any given country (i.e. at time t_0_; see Table 1). We define this rough proxy measure as *inherent salience*. It switches from 0 to 1 at time t_0_, and switches back to 0 as usual when binary closure takes effect. Model II of Table A4 tests this measure of inherent salience, compared with Model I, our preferred specification. We find that the coefficient is negative as expected, although not statistically significant. Closure is still negative and significant, but the effect size is reduced in this more approximate framework. Model III equalizes for epidemic length by using only the first 50 days of data for each country, to avoid countries with longer epidemics being more heavily weighted in the model. Compared to Model I we find that the coefficients are still significant and have roughly the same magnitude, suggesting that any country weighting effect does not introduce a bias.
